# Current Prostheses for Transcatheter Heart Valve Replacement: A Technical and Clinical Review 

**DOI:** 10.31083/j.rcm2308257

**Published:** 2022-07-20

**Authors:** Piotr Nikodem Rudziński, Markus Mach, Christoph Gross, Marco Russo, Paul Werner, Iuliana Coti, Sabine Scherzer, Martin Andreas

**Affiliations:** ^1^Department of Coronary and Structural Heart Diseases, National Institute of Cardiology, 04-628 Warsaw, Poland; ^2^Department of Cardiac Surgery, Medical University of Vienna, 1090 Vienna, Austria; ^3^Department of Cardiac Surgery and Heart Transplantation, San Camillo Forlanini Hospital, 00152 Rome, Italy

**Keywords:** transcatheter heart valve, transcatheter aortic valve implantation, transcatheter aortic valve replacement, interventional cardiology

## Abstract

Transcatheter aortic valve replacement (TAVR) has become a cornerstone in today’s treatment of aortic stenosis. Modern transcatheter prostheses are continuously evolving and each one features different design traits. In this review, the authors provide insight in the technical differences of current prostheses and TAVR related clinical decision pathways, preferably useful for the beginners but also for advanced operators. Additionally, procedural considerations and comparative outcomes of the prostheses are discussed. In doing so, the authors aim to facilitate the choice of the ideal transcatheter valve procedure for each individual.

## 1. Introduction

Transcatheter heart valve procedures for treating patients experiencing aortic valve stenosis (AS) are constantly improving. Transcatheter prostheses are the result of more than 50 years of valve development and can now be used to perform surgical-like procedures involving endovascular instruments without the need for cardiopulmonary bypass and cardioplegia [1]. Since its first clinical use by Alain Cribier in 2002 [2], this rapidly innovating procedure has transformed the clinical use of transcatheter aortic valve replacement (TAVR). In contrast to the early stages, when its use was limited to high-risk and inoperable patients, the indication is currently widening toward elderly, low- and intermediate-risk patients.

## 2. Current Guidelines

The promising results of recent large clinical trials helped to change indications and international guidelines. In particular, the PARTNER-III trial investigated the safety and effectiveness of the Edwards Sapien 3 heart valve in a cohort of low-risk patients affected by aortic stenosis and showed superiority of TAVR when compared to surgical aortic valve replacement (SAVR) in terms of a composite endpoint of death, rehospitalization for valve-related events or stroke (8.5 vs. 15.1%) at 12 months [3]. Similar results were addressed in the Evolut Low Risk Trial [4], which reported comparable outcomes of TAVR with the self-expandable Medtronic Evolut prosthesis when compared with SAVR for a primary composite endpoint of death or disabling stroke at 24 months (5.3 vs. 6.7%). The results of the two trials, which are similar in design and patient population involved, provided evidence for updating international guidelines. The EACTS/ESC guidelines indicated TAVR as a class I procedure beyond the age of 75 years, despite anatomical characteristics and patient informed decisions having to be included. The ACC/AHA valvular guidelines recommend TAVR in patients beyond the age of 80 years of any surgical risk category, and SAVR is favored in patients below the age of 65 years. Interestingly, TAVR or SAVR can be considered equally for symptomatic patients within the age range of 65 and 80 years [5, 6].

However, caution is warranted due to several unanswered questions. A high number of patients were excluded from these trials (bicuspid valves, severe calcification, low coronary ostia or unfavorable groin access), and the results cannot be extrapolated to patient populations not matching the patients investigated in these trials [7]. Furthermore, the incidence of permanent pacemaker implantation at one year (7.3% after Sapien 3 and 19.4% after Evolut implantation) and the rate of moderate or higher para-valvular leaks (PVL) are a matter of concern [8]. Most importantly, long-term durability due to structural deterioration and nonstructural valve dysfunction is a potential limiting factor for this treatment in a younger patient population. Therefore, life expectancy must be considered when treatment options are discussed. The 10-year follow-up of the present study as well as the results of an ongoing clinical trial, such as the EARLY TAVR and the UNLOAD trials, which investigate the role of TAVR in patients with asymptomatic AS and advanced heart failure, are presumed to resolve some of the controversial issues.

## 3. TAVR Prostheses

**Fig. 1.**
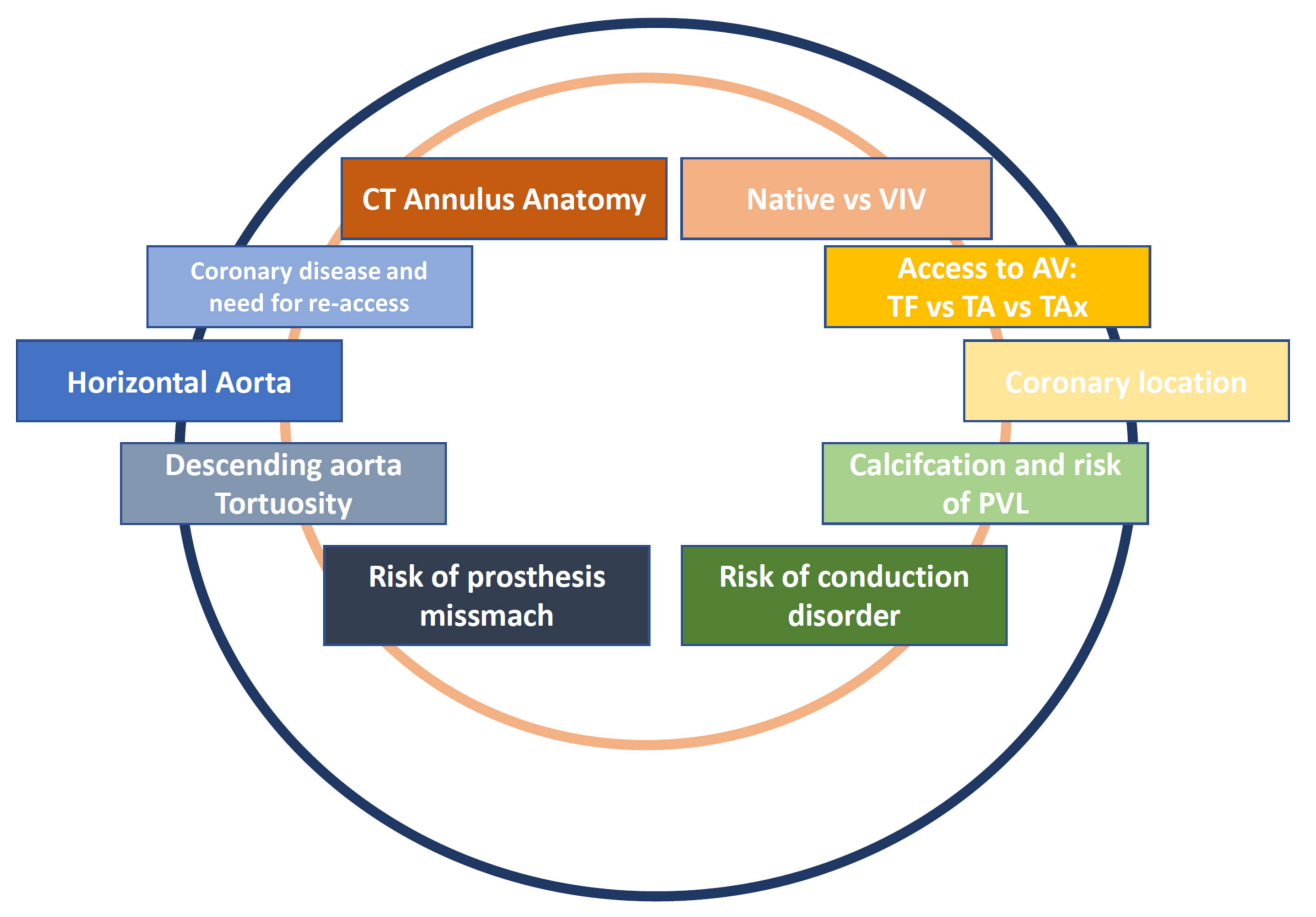
**Current clinical considerations and challenges in TAVR affecting patient evaluation, valve implantation and postoperative care**.

Several TAVR prostheses are currently available on the market, and patient selection criteria and device selection represent a challenge in daily clinical practice (Fig. 1). We herein present a current review with the aim of summarizing the state of the art of TAVR therapies to guide mainly those interventionalists who are not experts in this particular field. Our specific focus lays on device characteristics, differences and similarities, as well as patient selection and decision-making processes for patient- and anatomy-tailored valve implantation (Table 1).

**Table 1.** **Specifications of current TAVR prostheses and delivery systems**.

Company product	Frame material	Deployment	Leaflet material	Leaflet position	Valve size in mm	Annular dimensions	Access site(s)	Retrievable/Repositionable	Minimal vessel diameter	CE/FDA approval
Abbott - Navitor	Nitinol	Self-expanding	Bovine	Intra-annular	19–27 mm	19–27 mm	TF	Fully/Yes	5.0 mm	Yes (pending for TA)/Pending
Boston Scientific - Acurate neo2	Nitinol	Self-expanding	Porcine	Supra-annular	23, 25, 27 mm†	23, 25, 27 mm†	TF, TA	No/Yes	5.5 mm: 14F iSleeve	Yes/No
Edwards Lifesciences- Sapien XT	Cobalt chromium	Balloon-expanding	Bovine	Intra-annular	16–29 mm	16–29 mm	TF, TA, DA	No/No	6.0 mm with 16F Novaflex 3	Yes/Yes
									6.8 mm with 18F Novaflex 3	
									7.0 mm with 20F Novaflex 3	
Edwards Lifesciences- Sapien 3	Cobalt chromium	Balloon-expanding	Bovine	Intra-annular	18.6–29.5 mm‡	18.6–29.5 mm‡	TF, TA, DA	No/No	5.0 mm with 14F eSheath + 20 mm valve	Yes/Yes
									5.5 mm with 14F eSheath	
									6.0 mm with 16F eSheath + 29 mm valve	
Edwards Lifesciences- Sapien 3 Ultra	Cobalt chromium	Balloon-expanding	Bovine	Intra-annular	18.6–26.4 mm‡	18.6–26.4 mm‡	TF, TA, DA	No/No	5.5 mm with14F Axela (6.0 mm in TAx access)	Yes/Yes
Medtronic - CoreValve	Nitinol	Self-expanding	Porcine	Supra-annular	18–29 mm	18–29 mm	TF, TS, DA	No/Yes	6 mm	Yes/Yes
Medtronic-CoreValve Evolut R	Nitinol	Self-expanding	Porcine	Supra-annular	18–30 mm	18–30 mm	TF, TS, DA	No/Yes	5.0 mm with 14F In-Line Sheath	Yes/Yes
									5.5 mm with 16F In-Line Sheath	
Medtronic -CoreValve Evolut Pro	Nitinol	Self-expanding	Porcine	Supra-annular	18–30 mm	18–30 mm	TF, TS, DA	Partially/Yes	5.0 mm with 14F In-Line Sheath	Yes/Yes
									6.0 mm with 18F In-Line Sheath	
NVT AG - Allegra valve	Nitinol	Self-expanding	Bovine	Supra-annular	19–28 mm	19–28 mm	TF	Partially/Yes	6.0 mm	Yes/No

TF only; † perimeter derived diameter; ‡ area derived diameter. Abbreviations: DA, direct aortic access; TA, Transapical access; TF, Transfemoral access; TS, Transsubclavian/axillary access.

**Fig. 2.**
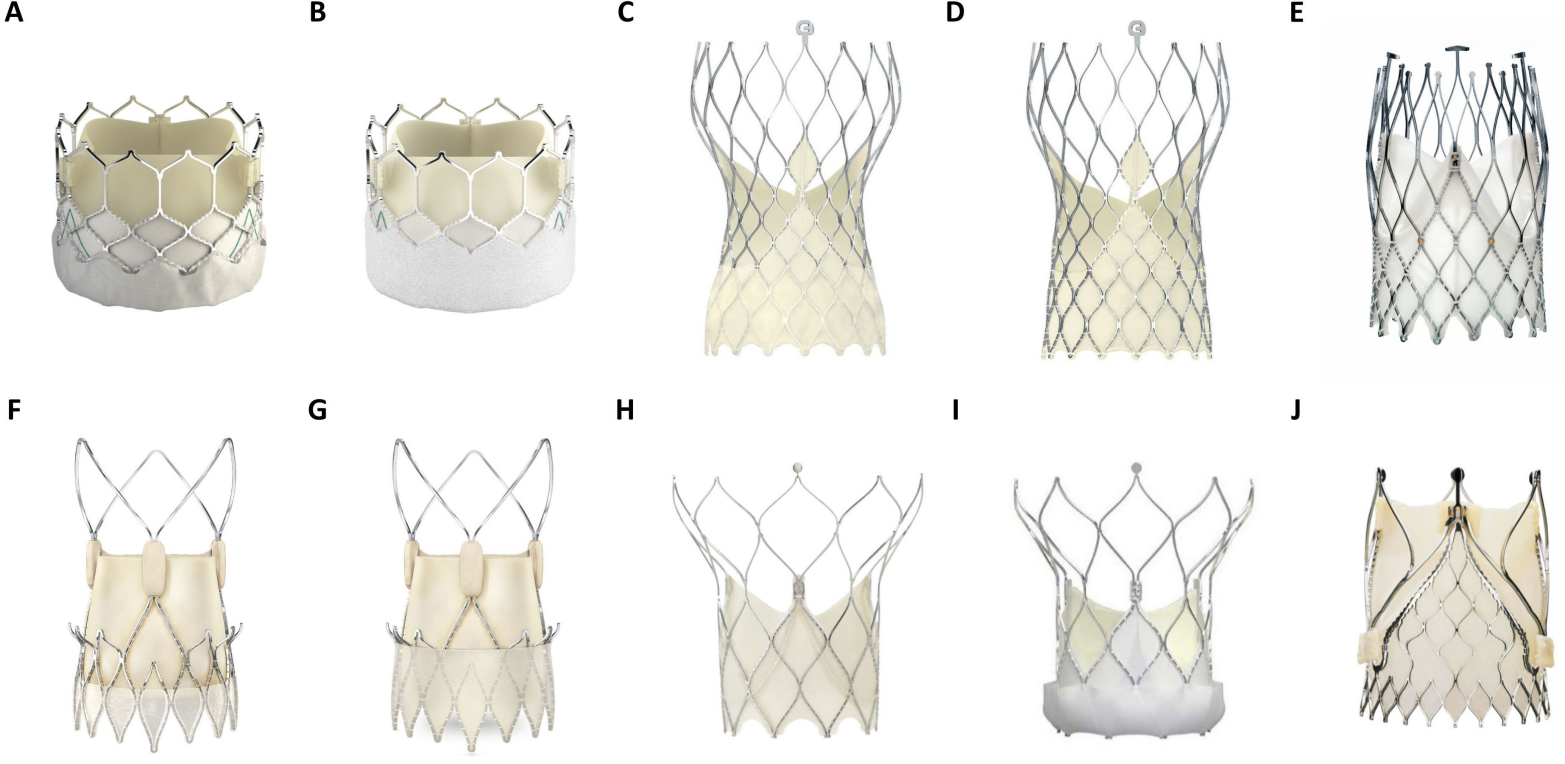
**Overview of several balloon (BEV)- and self expandable (SEV) TAVR prostheses in current clinical use**. (A) Edwards Lifesciences Sapien 3 (BEV). (B) Edwards Lifesciences Sapien 3 Ultra (BEV). (C) Medtronic Evolut Pro (SEV). (D) Medtronic Evlolut R (SEV). (E) NewValveTechnology Allegra (SEV). (F) Boston Scientific Accurate Neo (SEV). (G) Boston Scientific Accurate Neo 2 (SEV). (H) Abbott Portico (SEV). (I) Abbott Navitor (SEV). (J) JenaValve Trilogy (SEV). (Material provided courtesy of Edwards Lifesciences, Medtronic, NewValve Technology, Boston Scientific, Abbott and JenaValve Technology GmbH. All rights are reserved by each company. © 2022 Boston Scientific Corporation or its affiliates. All rights reserved.© February 2022 Medtronic. Portico and Navitor are trademarks of Abbott or its related companies. Reproduced with permission of Abbott, © 2022. All rights reserved. Used with the permission of Medtronic).

Transcatheter heart valves (THVs) comprise inherent design differences in stent frame, expansion mode and leaflet characteristics (Fig. 2). These specifications influence paravalvular sealing, hemodynamic function and periprocedural outcomes. THVs are categorized as balloon-expandable valves (BEVs), self-expanding valves (SEVs) and mechanically expanded valves (MEVs). Most of the currently available aortic THVs have been modelled after the original designs of the first-generation THVs, the SAPIEN (Edwards Lifesciences LLC, Irvine, CA, USA) for BEVs and CORE-VALVE (Medtronic, Minneapolis, MN, USA) for SEVs. Therefore, within the categorized groups (BEV or SEV), similarities in stent design, valve loading, implantation procedure and valve deployment exist.

Generally, the design of stents for THVs has progressed toward smaller prosthesis profiles, resulting in reduced diameters when compressed, recapturable and redeployable THVs with improved anchoring to prevent valve migration and paravalvular leaks.

## 4. Stent Design and Material

The stent frame must have the mechanical properties required to exert enough radial force to prevent prosthesis migration and to maintain valve orifice patency as well as tolerate compression into smaller delivery systems. For the SAPIEN device family (BEVs), changes in stent frame material from the initial stainless-steel-based frame of the SAPIEN to a cobalt-chromium-based frame of the SAPIEN XT, SAPIEN 3 and SAPIEN 3 Ultra resulted in the use of smaller delivery systems due to the lower prosthesis profile [9]. Furthermore, the denser cell structures in the annulus region of the stent frame as well as wider top struts of the SAPIEN 3 and SAPIEN 3 Ultra allow better sealing around the annulus and better coronary access in the supra-annular frame [10].

For SEVs such as Evolut R, Evolut Pro, Evolut Pro+ (Medtronic, Minneapolis, MN, USA), ACURATE neo (Boston Scientific, Natick, MA, USA) and Allegra (New Valve Technology, Hechingen, Germany), the stent frame is made of Nitinol. Nitinol comprises superior elasticity and shape memory function and is composed of nearly equal parts of nickel and titanium [11]. Low temperatures or high strain result in structural reconfiguration (phase transformation) of Nitinol, making the stents malleable for loading onto the delivery system. The Evolut R prosthesis makes use of the shape memory effect by low temperature (e.g., 0 °C to 8 °C or 32 °F to 46 °F), whereas other SEVs make use of the elastic material properties that allow valve loading at room temperature (Portico, ACURATE neo and Allegra). Similar to the BEVs mentioned above, the stent of the newer generation SEVs has a denser cell structure in the annulus region of the stent frame and wider top struts for better sealing around the annulus and improved coronary re-access.

The self-expanding nitinol stent frame of the ACURATE neo comprises an upper crown with the intent to provide tactile feedback during THV expansion. The prosthesis deployment process comprises two steps. First, during partial-unsheathing, the stabilization arms and the upper crown of the stent frame are released. Second, under rapid ventricular pacing (not mandatory), gentle maneuvering with the delivery device will bring the upper crown and the calcified leaflets in contact (tactile feedback), compressing the calcified tissue while at the same time adhering to the leaflets before the valve is fully expanded [12].

The JenaValve (JenaValve Technology GmbH, Munich, Germany) SEV is the only prosthesis with a temporary CE-mark for aortic regurgitation, in addition to the CE-mark for aortic valve stenosis. It was recently relaunched as a transfemoral device comprising of a stent frame with a clipping system that fixates the stent frame to the diseased aortic valve leaflets. This feature allows for prosthesis implantation even in minimally calcified aortic valves, in contrast to the aforementioned devices [13, 14].

Even though commercially unavailable, the Direct Flow Medical (Direct Flow Medical Inc, Santa Rosa, CA, USA) prosthesis is worth mentioning due to its nonmetallic stent frame. The following two-step process for valve expansion was performed. First, saline/contrast was used to expand the two rings of the prosthesis with eventual prosthesis repositioning. Second, following positioning, a quick-curing polymer was injected into the rings of the prosthesis replacing the saline/contrast. The double ring design of the prosthesis was intended to create a tight seal around the annulus [15].

## 5. Radial Force

Accurate sizing of the prosthesis stent according to the patient’s anatomy is critical to ensure proper anchoring [16]. For almost all THVs, prosthesis anchoring relies on the existing forces due to the use of oversized THVs compared to the native landing zone diameter. This radial force between the stent frame of the prosthesis and the surrounding anatomy must be sufficient to anchor the THV. Prosthesis oversizing and radial force have a direct impact on valve performance and procedure outcome.

**Fig. 3.**
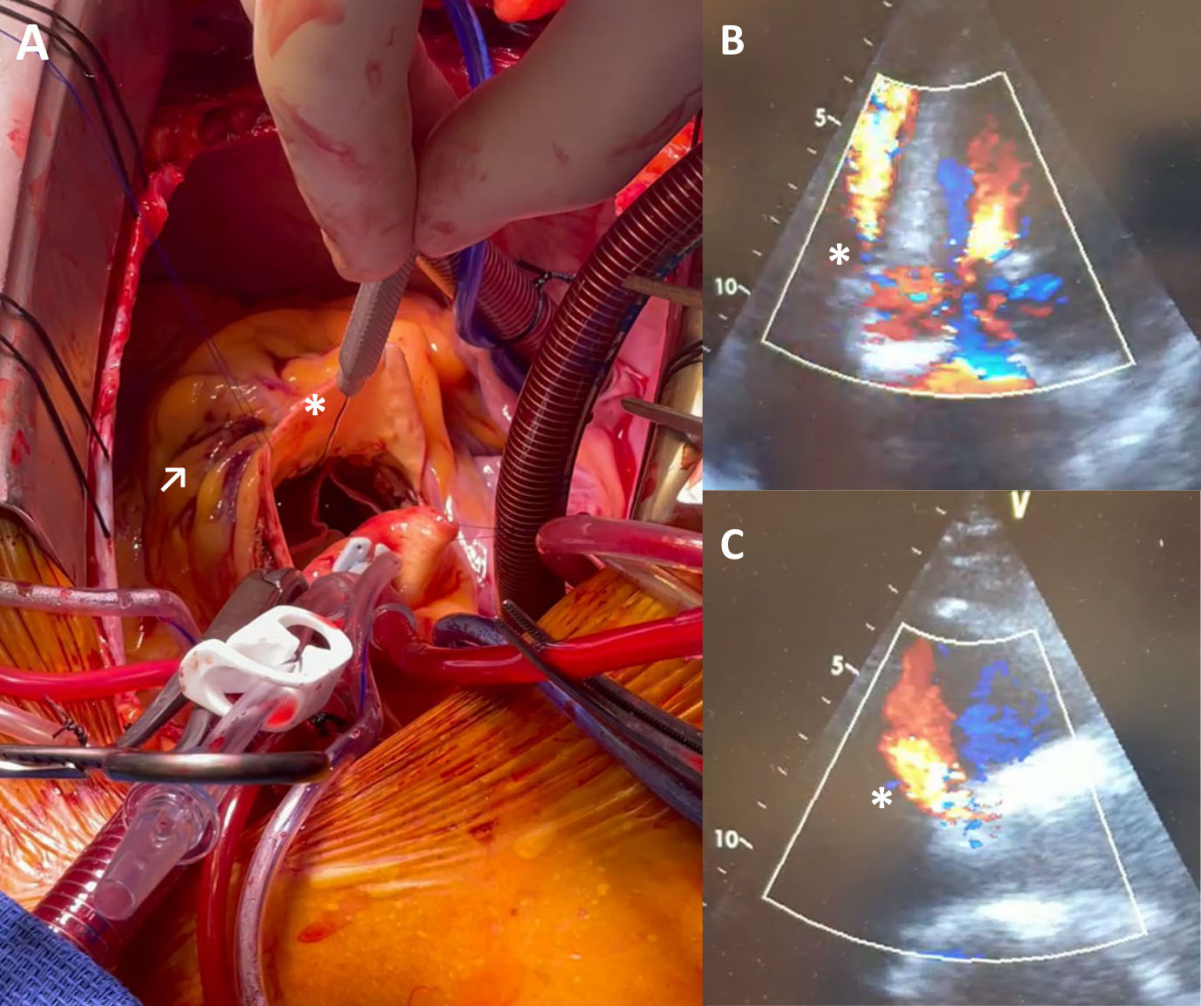
**Covered annular rupture with associated aorto-ventricular defect after implantation of a BEV following borderline oversizing**. (A) Surgical situs with introduction of a probe (*) in the RVOT from the aorta and corresponding epicardial hematoma (↗). (B) Four-chamber echocardiographic view after TAVR implantation with a high velocity jet (*) during diastole originating from the aorta until the right ventricular apex. (C) Regurgitation jet (*) in three-chamber view.

Insufficient oversizing of the THV can lead to paravalvular leakage, prosthesis embolism, limited orifice circularity or valve migration into the left ventricle during diastole. Prosthesis oversizing can lead to annulus rupture and conduction disturbances (Fig. 3). The resulting degree of oversizing after THV implantation depends on the diameter mismatch of the prosthesis and the native landing zone diameter as well as on the elasticity and stiffness of the surrounding anatomy together with the mechanical characteristics of the THV [17]. Therefore, choosing the optimal size of the THV in clinical practice can be challenging [18].

Oversizing strategies should be different according to the stent frame material due to their different mechanical interactions with the anatomy [18]. All SEVs comprise nitinol stent frames, whereas BEVs use cobalt-chromium for the Sapien XT, Sapien 3 and Sapien 3 Ultra. At valve deployment, SEVs spontaneously expand and strive for their full expanded shapes. In contrast, BEVs do not comprise the elastic properties of SEVs and are plastically deformed (irreversible) by the expansion of the balloon. In addition to possible recoil after balloon expansion, BEVs remain rigid. Furthermore, as clinically shown, BEVs maintain a high degree of orifice circularity after implantation in an oval annulus [19]. On the other hand, SEVs have the ability to change diameter according to the heart cycle and consequential anatomical dynamics [20].

The anchoring ability of the THV to the anatomical landing zone by the radial force is accompanied by other functionalities in some devices. For the Accurate Neo, the upper crowns may improve prosthesis anchoring and orientation. Due to the clips of the Jena Valve stent frame connecting the prosthesis to the native aortic valve leaflets, the device could be used even in noncalcified aortic valves. For SEVs, the flaired outflow segment of the stent frame provides additional anchoring support of the prosthesis to the patient’s anatomy with improved sealing capability. To reduce paravalvular leakage that could occur due to calcification or suboptimal valve orifice circularity, the prostheses’ frames for the SAPIEN 3, SAPIEN 3 Ultra, Navitor, Accurate Neo 2 and Evolut Pro+ comprise an outer-skirt at the lower part of the stent [21]. The pockets of the outer skirt of the SAPIEN 3 and the Navitor valve are designed to fill with retrograde clotting blood and thereby seal the gap between the native tissue and the THV. The other prostheses have an additional outer pericardial strip without pockets, which might have a limited sealing effect compared to the other system. Additionally, flaired inflow of the stent frame for some prostheses is intended to prevent paravalvular leakage.

## 6. Prosthesis Deployment

According to the technology (BE, SE), different deployment approaches are used. For BE THVs, deployment of the prosthesis is performed during rapid pacing to reduce left ventricular ejection and prevent changes in positioning of the delivery catheter, balloon catheter and THV during expansion. Positioning of BE THVs is performed in the crimped condition with the prostheses crimped on the delivery device, followed by a one-step prosthesis expansion.

SE THVs can be partially expanded to assess prosthesis position and recaptured for additional repositioning. The final step of this two-step approach is prosthesis deployment, for which rapid pacing may not be mandatory. For these THVs, a balloon might be used after deployment to reduce paravalvular leakage and improve orifice circularity and valve performance. The implantation technology of the Allegra allows positioning of the THV without interfering with the left ventricular outflow [22].

## 7. Leaflets

**Fig. 4.**
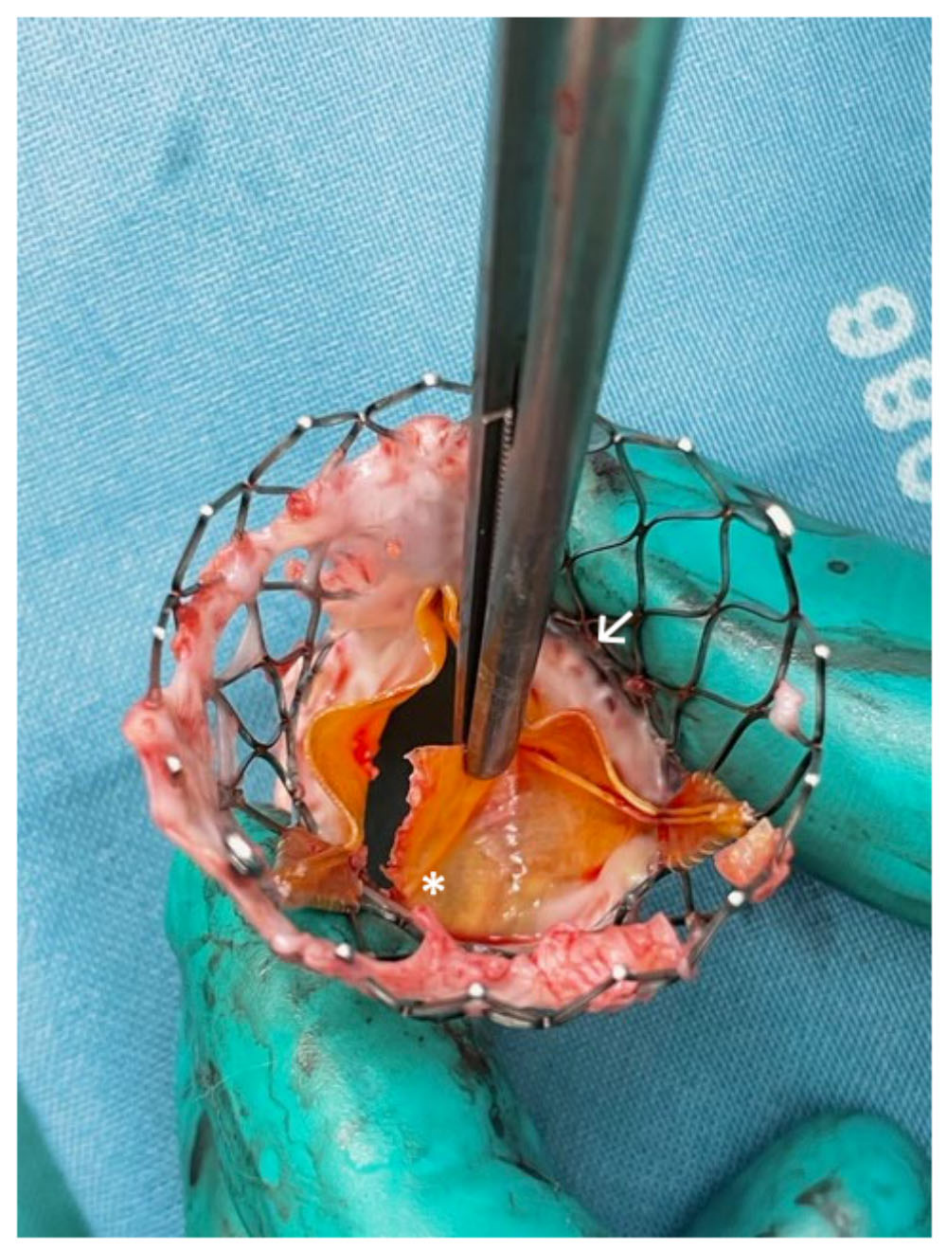
Surgically explanted SEV showing structural valve deterioration (SVD) in form of a commissural leaflet tear (*) which led to severe transvalvular regurgitation and beginning non-structural valve dysfunction (NSVD) with pannus ingrowth from the aortic side (↗) 2 approximately 24 months after implantation. This complication should be avoided by assuring the most appropriate prosthesis selection (EXPLANT-TAVR registry showed high median STS mortality rate of 5%).

Durability is a major concern with THVs, especially with their increasing use in younger and lower risk patients (Fig. 4). Tissue leaflets (bovine or porcine pericardium) have become the material of choice for the currently available THVs (see Table 1). Both tissue materials have different mechanical properties. Porcine pericardium tissue is stiffer and less extensible with similar tensile strength compared to bovine pericardium tissue [23]. Yap *et al*. [24] concluded in a review of clinical trials that bovine pericardium is superior compared to porcine pericardium in regard to complications and hemodynamic profile with comparable mortality, postoperative functional status and valve durability. Manufacturers of these bioprostheses employ dead tissues which are unable to grow, regenerate, remodel and repair themselves after damage.

Post-deployment leaflet injury occurs in bovine and porcine pericardial tissue valves, with eventual additional alterations due to crimping of BEVs [25]. Tissue materials for most bioprostheses are treated to reduce calcification of the prosthesis [13]. To allow proper valve coaptation for a less circular orifice, some SEVs are designed with the leaflets in the supra-annular position.

## 8. Implantation Concept

All of the clinical available THVs (see Table 1) are suited for the transfemoral access route, which is the access of choice for the majority of THV implantations. Multislice CT scans to screen potential vascular routes resulted in the reduction of vascular complications [26]. If femoral vascular access is not feasible, other strategies might be favored, such as the subclavian, carotid or transaortic route or transapical access [27, 28].

## 9. Neuroprotection

**Fig. 5.**
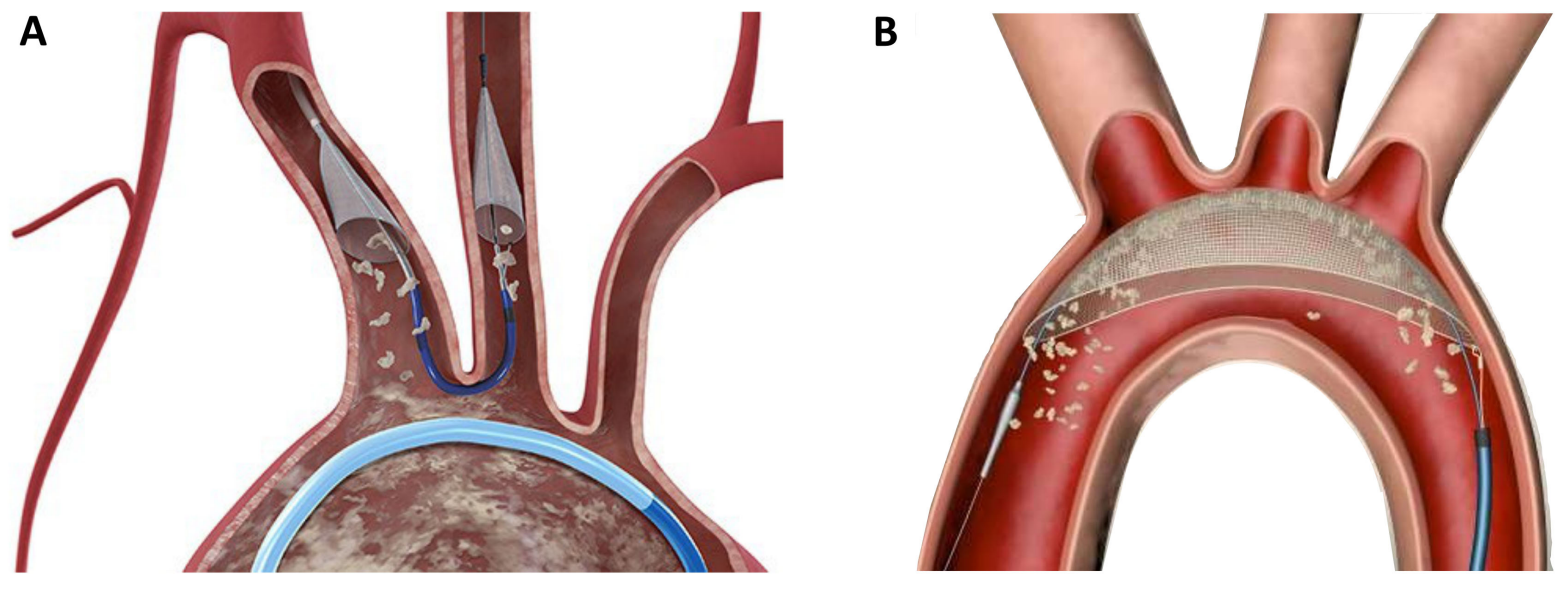
**Neuroprotection devices in current clinical use**. (A) The Sentinel device (Boston Scientific) is a filter device which is placed in the left carotid artery and the anonymate artery. (B) The TriGuard device (KeyStone Heart) is a deflection device which is positioned in the aortic arch.

Although TAVR can be performed with a favorable safety profile, the occurrence of stroke remains a serious complication. As the majority of periprocedural cerebral events are caused by calcific emboli, the concept of periinterventional neuroprotection emerged within the last years. Different devices were developed to prevent the migration of calcific particles in the supra-aortic vessels, which are mobilized during valve deployment or ballooning. The Sentinel device (Boston Scientific, Natick, MA, USA) consists of two filters which are placed in the left carotid artery and the anonymous artery via right radial access (6 Fr.) (Fig. 5A). The filters are designed to catch debris during the intervention, which is then removed at the end of the procedure during device retrieval. The TriGuard Device (KeyStone heart, Caesarea, Israel) is a based on a different protective mechanism; it is placed on the outer curvature of the aortic arch and designed to deflect debris further downstream in the descending aorta (Fig. 5B). Although large randomize trials are currently missing, clinical experience with these devices is promising.

## 10. Coronary Re-Access

The high prevalence of aortic stenosis and coronary artery disease (>60%) might be technically problematic with regard to coronary re-access after THV implantation (especially SEVs implantation causing THV crown neighboring with coronary ostia) [29]. It is recommended and preferable to perform percutaneous coronary intervention prior to TAVR procedure in order to avoid possible difficulties with coronary ostia cannulation after THV implantation [30]. However, taking into account that TAVR procedures would expand among younger groups of patients, the technical concerns regarding coronary re-access are of the increasing interest. Current studies aim to optimize THV implantation so that commissural alignment could be easily controlled during valve expansion. There are also trials that test transcatheter electrosurgical aortic leaflet laceration (such as BASILICA trial) to prevent coronary ostia from coronary leaflet obstruction [31]. Until now, there are no well-established patterns and techniques that guarantee non-problematic coronary re-access after TAVR procedure.

## 11. Current Evidence Comparing THVs

Several major postoperative adverse events, such as death, disabling stroke, para-valvular leakage, conduction disorders and permanent pacemaker implantation (PPI), major vascular complications, life-threatening bleeding, and acute kidney injury (AKI), have been largely reported in the current literature in the postoperative course of patients treated by means of TAVI [32]. There is still a limited number of randomized trials testing different THV clinical performances against each other, and outcomes of particular THVs as well as mid- and long-term follow-up data are limited. In the following literature review, we would like to concentrate on all existing randomized trials and support it with data from recent observational meta-analyses published up to 2021 to give an overview of the main clinical scenarios that can define a tailored approach in terms of device selection.

Historically, the first randomized trial comparing THVs in the group of high-risk patients with severe aortic stenosis was the CHOICE trial (121 patients received SAPIEN XT, and 120 patients received CoreValve) [33]. At 30 days, BEV resulted in a higher rate of device success (95.9% vs. 77.5%, *p *< 0.001) and a less frequent need for PPI (17.3% vs. 37.6%, *p* = 0.001). There was no significant difference in cardiovascular mortality (4.1% vs. 4.3%, *p* = 0.99) or in the number of strokes (5.8% vs. 2.6%, *p* = 0.33). The combined safety endpoints of death and main postoperative complications occurred in 18.2% of BEV patients and in 23.1% of SEV patients (*p* = 0.42). At one year, despite the greater device success rate with BEVs, there were still no significant differences in cardiovascular mortality (12.4% vs. 9.4%, *p* = 0.54) or the number of cerebrovascular events (9.1% vs. 3.4%, *p* = 0.11) with regard to SEVs [34].

In the SOLVE-TAVI trial, a newer generation of SAPIEN 3 (n = 222) and CoreValve Evolut R (n = 225) were compared with regard to a composite outcome of all-cause death, stroke, moderate-to-severe PVL and PPI. At 30 days, in 447 patients with severe symptomatic aortic stenosis, there were no significant differences among primary endpoints (26.1% vs. 27.2%, *p* = 0.02, for equivalence), whereas the potential for a higher stroke rate with BEV was observed (4.7% vs. 0.5%, *p* = 0.01).

The SCOPE I trial was a multicenter, randomized, noninferiority study testing the safety and efficacy of ACURATE Neo (SEV) in comparison to SAPIEN 3 in a group of high-risk patients with severe aortic stenosis [35]. At 20 sites, 739 patients were enrolled and allocated 1:1 to the SEV (n = 372) and BEV (n = 367) groups. At 30 days, there was no significant difference in the incidence of cardiovascular death (2.2% vs. 0.8%, *p* = 0.13) or the number of strokes (1.9% vs. 3.0%, *p* = 0.33). However, the primary endpoint (combination of VARC-2-derived endpoints of early safety and clinical efficacy at 30 days) occurred in 24% of patients from the SEV group and 16% of patients from the BEV group. The calculated absolute risk difference of 7.1% (with a one-sided upper 95% confidence limit of 12.0%) was lower than the prespecified noninferiority margin of 7.7%. Therefore, noninferiority of SEV was not achieved in the primary analysis (*p* = 0.42). Moreover, in the secondary analysis, the superiority of BEV was proven (*p* = 0.02), which was driven by less stage 2/3 AKI and less PVL. One-year follow-up is not available yet.

It can be concluded that the most thoroughly tested valve types are BEVs (mainly SAPIEN family valves - SAPIEN XT and SAPIEN 3) and SEVs (mainly CoreValve family valves - CoreValve Classic and Evolut R) at follow-up times of 30 days and 1 year. It was shown that implantation of BEV (while compared to SEV) is connected with the following: (1) Higher device success and lower PPI rates, (2) Similar cardiovascular mortality and the composite of safety and efficacy endpoints derived from VARC-2 criteria and (3) Higher incidence of stroke. However, in the largest propensity-matched analysis of observational studies comparing different valve types in TAVI (n = 12,381), it was reported that stroke was less frequent in BEV patients than in SEV patients [36]. Moreover, patients treated with BEVs had more major or life-threatening bleeding than patients treated with SEVs. However, 30-day mortality and the lower need for PPI in the case of BEV patients compared to SEV patients were consistent with previous randomized trials.

Importantly, despite enormous progress in THV design over the next years, for example: (1) stainless-steel frame in SAPIEN, (2) a cobalt-chromium alloy frame in SAPIEN XT, (3) a cobalt-chromium alloy frame and an adaptive external polyethylene terephthalate fabric seal in SAPIEN 3, frequencies of mild PVL and moderate prosthesis-patient mismatch did not decrease significantly and were accordingly of: (1) 38.0% and 30.0% in PARTNER-I, (2) 33.2% and 32.8% in PARTNER-II, (3) 28.8% and 29.4% in PARTNER-III [37, 38].

## 12. Conclusions

Transcatheter heart valve technology is continuously evolving as well as clinical indications and patient management strategies. Detailed knowledge of device differences and accurate patient selection are mandatory to improve short- and long-term results. Prosthesis selection according to anatomy and clinical features represents a key step for successful and durable treatment and must be critically included in the heart team discussion. Several issues, such as durability, bicuspid aortic valves, valve-in-valve procedures and coronary re-access, remain unclear and must be clarified before expanding the application of this technology to a younger patient population.
